# Multidimensional pain patterns in Parkinson’s disease: a longitudinal mobile health study

**DOI:** 10.1007/s00702-026-03250-9

**Published:** 2026-07-30

**Authors:** Eduardo Souza-Silva, Ana Carolina Ruver-Martins, Amanda Karoline Venceslau Cassenotte, Fabiana Noronha Dornelles, Marina Bueno Recchia, Carolina Franchi de Souza Sá, Filipe Carvalho Matheus, Samantha Cristiane Lopes, Rui Daniel Prediger

**Affiliations:** 1 Department of Pharmacology, Center of Biological Sciences, Universidade Federal de Santa Catarina, Florianópolis, CEP: 88040-900 Santa Catarina Brazil; 2 Department of Pharmaceutical Sciences, Health Sciences Center, Federal University of Santa Catarina, Florianópolis, Santa Catarina Brazil; 3 Faculty of Medicine, Research Center in Medical Sciences, Investigations in Health-NPCMed, Centro Universitário para o Desenvolvimento do Alto Vale do Itajaí-UNIDAVI, Jardim América, Rio do Sul, 89160-932 SC Brazil; 4 TechPain Scientific Research and Development, Florianópolis, Santa Catarina Brazil

**Keywords:** Parkinson’s disease, Chronic pain, Mobile health, Unsupervised learning, Pain distribution

## Abstract

Pain is a non-motor symptom of Parkinson’s disease (PD). In clinical practice, it is often reduced to single metrics, such as intensity, omitting important aspects of its complexity. Mobile health tools enable repeated recording and multidimensional analysis of pain. To examine how pain intensity, duration, and spatial distribution differentiate patient profiles using longitudinal mobile health data. We analyzed 20,971 daily records from 68 PD patients over 14 months. Patients reported pain intensity, episode duration, and pain location. Data were aggregated at the individual level to avoid pseudo-replication. Profiles were identified using unsupervised clustering (HDBSCAN with KMeans fallback). Associations between pain dimensions were assessed using Spearman correlation. Two profiles were identified: a high-burden group (*n* = 17) and a low-burden group (*n* = 50). One additional cluster (*n* = 1) was excluded due to instability. Differences between groups were greater for cumulative pain duration (6.7-fold) and spatial extent (9–11-fold) than for mean intensity (2.4-fold). Mean pain intensity was moderately correlated with cumulative duration (ρ = 0.66) and spatial extent (ρ = 0.60 anterior; ρ = 0.44 posterior), whereas mean episode duration showed weak and non-significant associations with other pain metrics. Recording frequency was not associated with cluster membership (*r* = 0.16, *p* = 0.20), and both groups showed sustained engagement. Pain intensity alone may not adequately reflect overall burden in PD. Considering temporal persistence and spatial distribution provides a more complete view of patient experience and supports a multidimensional approach to assessment.

## Introduction

Pain is a prevalent and clinically significant non-motor symptom in Parkinson’s disease (PD), a complex multisystem neurodegenerative disorder characterized by a broad spectrum of motor and non-motor manifestations (Poewe et al. 2017; Chaudhuri et al. 2006). It affects up to 85% of patients at all stages of the disease (Broen et al. 2012; Schneider et al. 2013) and is associated with reduced quality of life, impaired function, and increased use of health services (Chaudhuri and Schapira 2009; Martinez-Martin et al. 2011). Despite its clinical importance, pain in PD remains insufficiently characterized in clinical practice, especially regarding its temporal persistence and spatial distribution.

A major limitation lies in the reliance on cross-sectional assessments and unidimensional measures, normally using pain intensity as a reference. The pain in PD is dynamic, fluctuating over time involving multiple coexisting mechanisms, including musculoskeletal, neuropathic, and central components (Ford 2010; Sung et al. 2018), as well as temporal interactions with affective and contextual factors observed in longitudinal diary-based studies (Feldman et al. 1999; Shiffman et al. 2008). Episodic clinical assessments, therefore, may not capture the full temporal and spatial complexity of the symptom, which can lead to underestimation of the overall burden and inadequate management (Chaudhuri and Schapira 2009).

Mobile health technologies provide a complementary approach. They allow for the frequent collection of patient-reported data in the real world (Rajkomar et al. 2019; Bougea 2025), as demonstrated in large-scale smartphone-based studies in Parkinson’s disease (Bot et al. 2016). This longitudinal data makes it possible to recognize pain as a multidimensional and dynamic phenomenon, capturing patterns that are not accessible through conventional clinical assessments. Analytical approaches applied to these datasets can facilitate the identification of structured patterns among patients and support a more comprehensive characterization of symptom burden (Rajkomar et al. 2019).

However, previous studies, including those applying machine learning to pain assessment, have focused predominantly on intensity-based measures (Lötsch and Ultsch 2018; Zhang et al. 2023), which may potentially underrepresent other relevant dimensions, such as duration, temporal persistence, frequency, and spatial distribution. It is still unclear whether incorporating these additional dimensions provides incremental value over intensity alone in distinguishing clinically significant patient subgroups. Furthermore, few studies have explored patient-level aggregation strategies that explicitly account for temporal variability, avoiding pseudo-replication, a critical methodological issue in longitudinal symptom screening (Hurlbert 1984). In this study, our aim was to identify clinically interpretable pain profiles in PD patients using longitudinal mobile health data, and we further sought to determine how different dimensions of pain contribute to differentiation between patient subgroups. A better understanding of multidimensional pain patterns may improve clinical assessment and support more individualized management strategies in Parkinson’s disease.

## Methods

### Study design and population

This study is a secondary analysis of longitudinal symptom data collected in a clinical study conducted at the Federal University of Santa Catarina (Florianópolis, Brazil). The original study involved the prospective monitoring of patients with Parkinson’s disease using a mobile health platform for continuous symptom monitoring under controlled conditions. The original study followed the principles of the Declaration of Helsinki and Good Clinical Practice guidelines and was approved by the Research Ethics Committees of the State University of Western Paraná (Unioeste; CAAE 59839722.1.0000.0107) and the Federal University of Santa Catarina (UFSC; CAAE 59839722.1.3001.0121). All participants provided free and informed written consent. This study focused exclusively on observational symptom data collected through the mobile health application and does not address the objectives of the original study. Thus, this work should be interpreted as a secondary observational analysis of prospectively collected data. The data were analyzed retrospectively from patients with Parkinson’s disease (PD) who used a mobile health app for daily symptom monitoring, from December 2023 to February 2025 (14 months). Inclusion criteria were: diagnosis of idiopathic PD, age ≥ 50 years, and participation in the prospective clinical study, which included longitudinal symptom monitoring using the mobile health application. No predefined minimum level of app use was established as an eligibility criterion for this secondary analysis. The age threshold was selected because idiopathic Parkinson’s disease predominantly affects older adults, and this criterion was adopted to obtain a clinically homogeneous study population. All data were anonymized before analysis. The final cohort included 68 patients, contributing 20,971 daily records.

### Variables and data collection

Data were collected through a dedicated mobile health app (TechPain Monitor), designed for daily symptom monitoring in patients with chronic pain conditions, consistent with prior studies using smartphone-based platforms to generate digital outcome measures in Parkinson’s disease (Lipsmeier et al. 2018). The platform enabled the collection of patient-reported data, including pain intensity (numerical rating scale from 0 to 10, NRS), episode duration (minutes), and spatial distribution using digital body mapping with anterior and posterior views. The body mapping interface used a standardized coordinate space and resolution for all participants. Additional variables included the use of medications across multiple therapeutic classes, encompassing antiparkinsonian drugs, analgesics, nonsteroidal anti-inflammatory drugs (NSAIDs), antihypertensives, and other concomitant treatments. Information on sleep duration was collected using categorical self-reported responses (< 2 h, 2–5 h, 5–7 h, ≥ 8 h). The assessment of symptoms was based on a predefined list of items within the application, including motor symptoms (tremor, rigidity, bradykinesia, involuntary movements, and gait disturbances) and non-motor symptoms, encompassing neurological manifestations (cognitive slowness, confusion, sensory alterations, and speech alterations), sleep-related symptoms (insomnia, fragmented sleep, daytime sleepiness, and nightmares), psychiatric symptoms (anxiety, mood swings, agitation, stress, and hallucinations), gastrointestinal symptoms (nausea, vomiting, constipation, reflux, abdominal discomfort, and difficulty swallowing), cardiovascular symptoms (blood pressure fluctuations, chest discomfort, and syncope), respiratory symptoms (dyspnea, cough, respiratory irregularities, and choking episodes), urinary symptoms (incontinence and nocturia), and dermatological or mucosal symptoms (skin changes, pruritus, and xerostomia). Patients could also report symptoms using free text. All reported symptoms were processed, and free-text entries were standardized through approximate matching and grouped into clinically relevant categories. An explicit asymptomatic category was included to capture reporting periods without symptoms. For each daily entry, symptoms were coded as binary indicators of presence or absence. Subsequently, all symptoms were mapped onto predefined clinical domains (neurological, balance and gait, sleep-related, psychiatric, gastrointestinal, cardiovascular, respiratory, urinary, and dermatological) to derive domain-level variables for analysis. Domain assignment followed the clinical grouping of motor and non-motor manifestations (e.g., neurological domain including motor and sensorimotor features; balance/gait domain capturing postural instability and locomotion-related symptoms). The classification of symptoms into discrete domains remains heterogeneous in the literature, without a universally accepted framework, reflecting the multidimensional and variable nature of symptom presentation in Parkinson’s disease (Chaudhuri et al. 2006; Martinez-Martin et al. 2011). Therefore, the domains in this study were defined based on clinically established groupings and used for subsequent analyses.

### Construction and aggregation of features

Since the dataset consisted of repeated observations for each individual, daily records were aggregated at the patient level for subsequent analyses, avoiding pseudo-replication (Hurlbert 1984; Stone et al. 2012; Billot et al. 2025). A comprehensive set of features was derived from longitudinal records to represent multiple dimensions of pain and clinical status. These included measures of central tendency (e.g., mean pain intensity, mean episode duration), cumulative burden (e.g., total pain duration), spatial pain area measures (anterior and posterior) derived from digital body maps, as well as symptom-based metrics and treatment-related variables consistent with established approaches for deriving outcome measures from momentary assessment data (Stone et al. 2012). Pain area was calculated from digital body maps using a standardized model with fixed resolution, allowing direct comparison of spatial extent across patients. This approach provides information not captured by intensity alone (Billot et al. 2025; Ciampi de Andrade et al. 2023). Overall pain burden was operationally defined as cumulative pain duration across the observation period, representing the recorded persistence of pain rather than a direct measure of absolute exposure (Von Korff et al. 1992). Pain crises were defined as episodes with intensity ≥ 7 on the numerical rating scale (Dworkin et al. 2005), a threshold commonly used to indicate severe pain in chronic pain research (Dimitriadis et al. 2024; Dworkin et al. 2005). Pain crisis-related variables were summarized using three complementary metrics: total number of crises, crisis frequency (number of events per 7-day period, averaged across the observation period), and mean days between crises (average interval between consecutive events). These variables were treated as indicators of episodic pain dynamics rather than independent dimensions of overall pain burden. Medication-related variables were summarized using three complementary metrics: medication frequency, the number of drug classes, and a therapeutic complexity score. Medication frequency was defined as the total number of medication reports recorded across the observation period. Medication use was summarized according to pharmacological classes to provide a stable representation of longitudinal treatment patterns because medication regimens could change throughout routine clinical follow-up. The number of drug classes reflected the diversity of pharmacological treatments used by each patient. The therapeutic complexity score was calculated as √(N × D), where N is the number of distinct pharmacological classes used by the patient, and D is the sum of total daily doses (in mg) weighted by class-specific coefficients ranging from 0.5 (e.g., phytotherapics) to 2.0 (e.g., anticonvulsants and barbiturates). Weights were assigned based on the authors’ assessment of relative treatment burden, inspired by the Drug Burden Index (Hilmer et al. 2007). The square root transformation was applied to dampen exponential growth. The resulting score is a unitless composite measure, with higher values indicating greater therapeutic complexity. The final score was obtained by summing the weighted contributions across all classes used by each patient. Sleep adequacy was defined as ≥ 6 h of sleep per night (binary), based on self-reported categorical sleep duration responses which were converted into approximate hourly values for analysis. Symptom-related variables were operationalized as quantitative measures, including the total number of symptoms per record, the number of active symptom domains, and domain-specific symptom counts (e.g., neurological, sleep-related, equilibrium and gait, psychiatric, gastrointestinal, and other domains). Although multiple symptom domains were initially defined, only those with sufficient frequency (> 10%) of patients were included in subsequent analyses. Domain-specific symptom variables were computed as the frequency of symptom reports within each domain across the observation period. The number of active symptom domains was defined as the count of distinct symptom categories reported per daily record and subsequently aggregated at the patient level. These variables captured the frequency and distribution of symptoms across domains over time. An “asymptomatic” category was defined based on patient-reported entries indicating absence of symptoms (e.g., “no symptoms”, “no new symptoms”, or equivalent responses). This variable reflects symptom-free reporting periods within individuals rather than absence of disease and was treated as a distinct symptom domain. To assess the contribution of each variable to group separation, we calculated the amplitude (difference between maximum and minimum values) between clusters. Variables with larger amplitudes were interpreted as showing greater between-cluster separation, providing a descriptive indication of their relative contribution to group differentiation rather than a formal measure of feature importance (Jain 2010).

### Clustering analysis

Unsupervised clustering was performed on patient-level feature vectors using HDBSCAN (Python, hdbscan library, version 0.8.33), a density-based clustering algorithm that identifies clusters of varying density and labels low-density points as noise (McInnes et al. 2017). We used a minimum cluster size of 2, minimum samples of 1, Euclidean distance, and excess-of-mass cluster selection, as originally described for the method (Campello et al. 2013; McInnes et al. 2017). When more than 10% of observations were labeled as noise by HDBSCAN, indicating an unstable clustering solution with substantial data fragmentation, a predefined KMeans fallback was applied to obtain more stable and clinically interpretable groupings (Hurlbert 1984). This step was used to improve interpretability in a small-sample setting, where density-based methods may produce fragmented solutions. Clustering was used as an exploratory approach to identify patterns in multidimensional features rather than to define fixed clinical subtypes. The number of clusters (k = 3) was selected to allow full data partitioning and to minimize information loss due to noise classification in the density-based solution, while preserving clinically interpretable subgroup structure, consistent with standard practices in exploratory clustering (Rousseeuw 1987). Clustering was performed without predefined labels, and groups were interpreted post hoc based on their clinical characteristics. Clusters with fewer than five patients were considered unstable and excluded from comparative analyses. To assess potential engagement bias, we examined the association between the number of active recording days per patient and cluster membership using point-biserial correlation.

### Statistical analysis

Comparisons between identified patient profiles were performed using independent t-tests for continuous variables. Categorical variables were compared using chi-square tests or Fisher’s exact test, as appropriate. Spearman rank correlation coefficients were used to assess associations between pain metrics (intensity, duration, and spatial extent) and clinical variables, including symptom domains, medication-related measures, and crisis-related variables. Correlation analyses were performed using patient-level aggregated data. Point-biserial correlation was used to assess engagement bias by examining the relationship between days active and cluster membership. Given the exploratory nature of this hypothesis-generating study, statistical significance was set at a two-sided alpha level of 0.05 without adjustment for multiple comparisons (Bender and Lange 2001). All analyses were performed using Python version (3.12.8).

## Results

### Patient characteristics

The study population consisted of 68 patients with a mean age of 68.2 ± 8.4 years (range 52–84 years). Of these, 38% (*n* = 26) were female.

### Identification of patient profiles

**Fig. 1:**
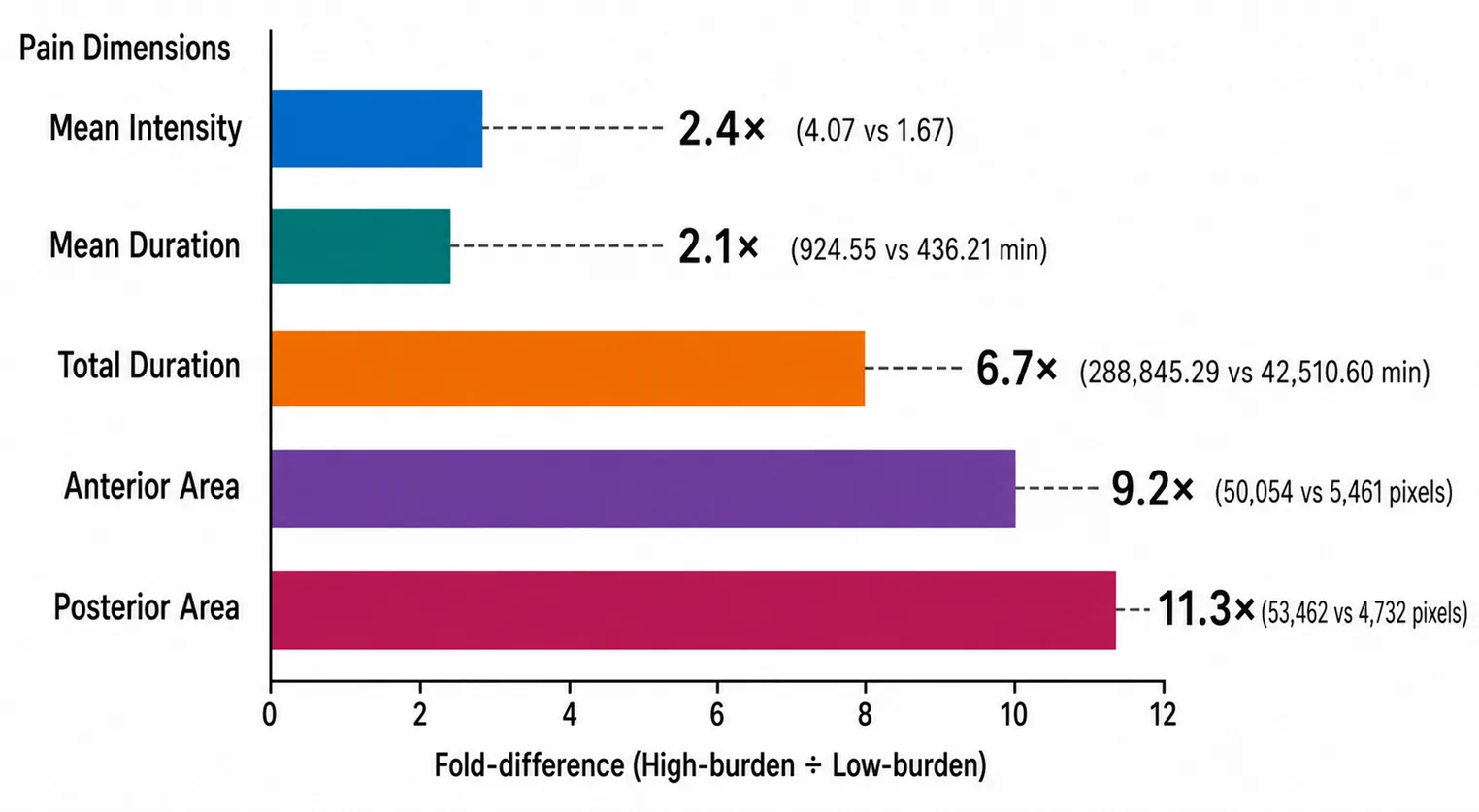
Relative differences between high-burden and low-burden patient profiles across pain dimensions. Cumulative pain duration and spatial extent demonstrated substantially greater differences between groups compared to mean pain intensity, supporting their stronger contribution to between-group differences

Initial cluster analysis using HDBSCAN identified two groups (*n* = 36), with 32 patients (47.1%) classified as noise. Following the predefined analytical workflow, a three-group KMeans algorithm was then applied, which yielded more stable and clinically interpretable subgroups. The KMeans clustering identified three patient groups. One group contained only one patient and was excluded from comparative analyses. The two remaining clinically interpretable profiles comprised a high pain burden group (*n* = 17) and a low pain burden group (*n* = 50). Both groups demonstrated substantial engagement throughout the 14-month study period. Patients in the high pain burden group recorded an average of 156.8 ± 92.4 active recording days, while those in the low pain burden group recorded on 112.3 ± 85.1 active recording days. The point-biserial correlation between the days of activity recording and participation in the group was weak and not significant (*r* = 0.16, *p* = 0.20). The high pain burden profile was characterized by consistently higher values across multiple pain dimensions. No significant differences were observed between groups regarding age or sex distribution (Table 1). The mean pain intensity was higher in the high pain burden group (4.07 ± 1.2) compared to the low-burden group (1.67 ± 0.9; *p* < 0.001), representing a 2.4-fold difference. However, the observed differences were more pronounced for cumulative and spatial measures. The total duration of pain showed a 6.7-fold difference between the groups (*p* < 0.001). The spatial extent was greater in the high pain load group, with an anterior pain area of 50,054 ± 28,941 pixels compared to 5461 ± 4203 pixels (a 9.2-fold difference; *p* < 0.001) and a posterior pain area of 53,462 ± 31,207 pixels compared to 4732 ± 3891 pixels (an 11.3-fold difference; *p* < 0.001). Maximum pain intensity was also higher in the high pain burden group (8.11 ± 1.3 vs. 6.76 ± 1.8; *p* = 0.004). Medication complexity was higher in the high pain burden group, with a greater number of drug classes (6.6 ± 1.8 vs. 4.7 ± 1.9; *p* < 0.001) and higher therapeutic complexity scores (1481 ± 412 vs. 714 ± 298; *p* < 0.001). Although the use of antiparkinson drugs and conventional analgesics was comparable between groups, tricyclic antidepressants were more frequently used in the high pain burden group (24% vs. 2%; *p* = 0.01). These patterns are summarized in Fig. 1, which highlights the dominant contribution of cumulative pain duration and spatial extent to group differentiation, compared with mean pain intensity. Symptom burden also increased, with a higher total number of symptoms (3.58 ± 1.2 vs. 2.24 ± 0.9; *p* < 0.001) and a greater number of active symptom clusters (1.97 ± 0.6 vs. 1.21 ± 0.5; *p* < 0.001). The differences observed were greater in the neurological and sleep-related domains including motor-related features as tremor and rigidity and sleep-related domains, where 89% and 100% of patients with high symptom burden reported at least one symptom, respectively. Sleep adequacy did not differ significantly between groups (17% vs. 25%; *p* = 0.32), and no significant difference was observed in the distribution by age or sex. A complete summary of the demographic, pain, therapeutic, and symptom characteristics of both patient profiles is provided in Table 1.

**Table 1:** Demographic, pain, therapeutic, and symptom characteristics according to pain-burden profile

Characteristic	Low pain burden (*n* = 50)	High pain burden (*n* = 17)	*P* value
*Demographic profile*			
Age (years)	67.9 ± 8.3	69.9 ± 8.4	0.4
Female sex, *n* (%)	20 (40%)	5 (29.4%)	0.56
*Pain burden profile*			
Mean pain intensity	1.67± 0.9	4.07± 1.2	< 0.001
Cumulative pain duration (min)	42,510.6 ± 48,838.1	288,845.3 ± 112,955.0	< 0.001
Anterior pain area (pixels²)	5461 ± 4203	50,054 ± 28,941	< 0.001
Posterior pain area (pixels²)	4732 ± 3891	53,462 ± 31,207	< 0.001
*Medication profile*			
Number of medication classes	4.7 ± 1.9	6.6 ± 1.8	< 0.001
Therapeutic complexity score	714 ± 298	1481 ± 412	< 0.001
Antiparkinson drugs (%)	48 (96)	17 (100)	1
Analgesics (%)	21 (42)	7 (41)	1
NSAIDS (%)	20 (40)	6 (35)	0.78
Calcium-channel anticonvulsants (%)	6 (12)	4 (24)	0.24
Sodium-Channel anticonvulsants (%)	1 (2)	0 (0)	1
GABAergic anticonvulsants (%)	1 (2)	1 (2)	1
Tricyclic antidepressants (%)	1 (2)	4 (24)	0.01
SSRIs (%)	10 (20)	4 (24)	0.7
SNRIs (%)	7 (14)	3 (18)	0.7
Atypical antidepressants (%)	4 (8)	3 (18)	0.3
*Symptoms profile*			
Total number of symptoms	2.24 ± 0.9	3.58 ± 1.2	< 0.001
Active symptom domains	1.21 ± 0.5	1.97 ± 0.6	< 0.001

Categorical variables are presented as number of patients (% of participants within each group). Medication classes indicate the number and proportion of patients who used at least one drug from each pharmacological class during follow-up; because medication regimens could change over time, patients may be represented in more than one pharmacological class. Continuous variables are presented as mean ± standard deviation. *P* values were calculated using independent *t*-tests for continuous variables and the χ² test or Fisher's exact test for categorical variables, as appropriate

### Feature importance ranking for cluster differentiation

To quantitatively assess which dimensions of pain differentiated the patient profiles, we classified all characteristics by their amplitude (maximum-minimum value) between the high and low pain burden profiles. The ten main discriminative characteristics are presented in Table 2. The cumulative pain duration showed the greatest absolute amplitude (difference of 246,335 min between clusters), followed by the posterior pain area (48,730 pixels), anterior pain area (44,593 pixels), total medication frequency (15,521 occurrences) and therapeutic complexity score (818, composite metric). The average pain intensity ranked 10th among all discriminative variables (amplitude of 2.4 NRS units), with the first nine positions occupied by cumulative duration, spatial extent, and medication-related characteristics, indicating a comparatively smaller contribution of intensity-based measures to between-group differentiation.

**Table 2:** Top 10 features discriminating high-burden from low-burden profiles

Rank	Feature	Amplitude	Fold-difference
1	Cumulative pain duration (minutes)	246,335	6.7×
2	Posterior pain area (pixels)	48,730	11.3×
3	Anterior pain area (pixels)	44,593	9.2×
4	Total medication frequency	15,521	1.8×
5	Therapeutic complexity score	818	2.1×
6	7-day duration trend (minutes)	488	2.0×
7	Mean pain duration (last 7 days, minutes)	485	2.1×
8	Mean pain duration per episode (minutes)	454	2.0×
9	Median pain duration (minutes)	378	2.0×
10	Mean pain intensity (0–10 NRS)	2.4	2.4×

Amplitude represents the absolute between-cluster difference calculated from the aggregated patient-level features used in clustering. Fold-difference represents high-burden vs. low-burden ratio. The ranking is based on absolute magnitude, not fold difference

### Correlation analysis of pain dimensions

Mean pain intensity showed a moderate positive correlation with cumulative pain duration (ρ = 0.66, *p* < 0.001), as well as with anterior (ρ = 0.60, *p* < 0.001) and posterior spatial extent (ρ = 0.44, *p* < 0.001). The cumulative pain duration showed moderate correlations with mean duration (ρ = 0.54, *p* < 0.001), anterior spatial extent (ρ = 0.57, *p* < 0.001), and posterior spatial extent (ρ = 0.43, *p* < 0.001). Spatial extent measures were moderately correlated with each other (ρ = 0.50, *p* < 0.001). In contrast, mean pain duration per record showed weak and non-significant correlations with mean pain intensity (ρ = 0.15, *p* = 0.23), anterior (ρ = 0.17, *p* = 0.16) and posterior (ρ = 0.07, *p* = 0.55) spatial extent measures, indicating that average episode duration does not systematically vary with other pain metrics.

### Correlation of pain metrics with symptom variables

**Fig. 2:**
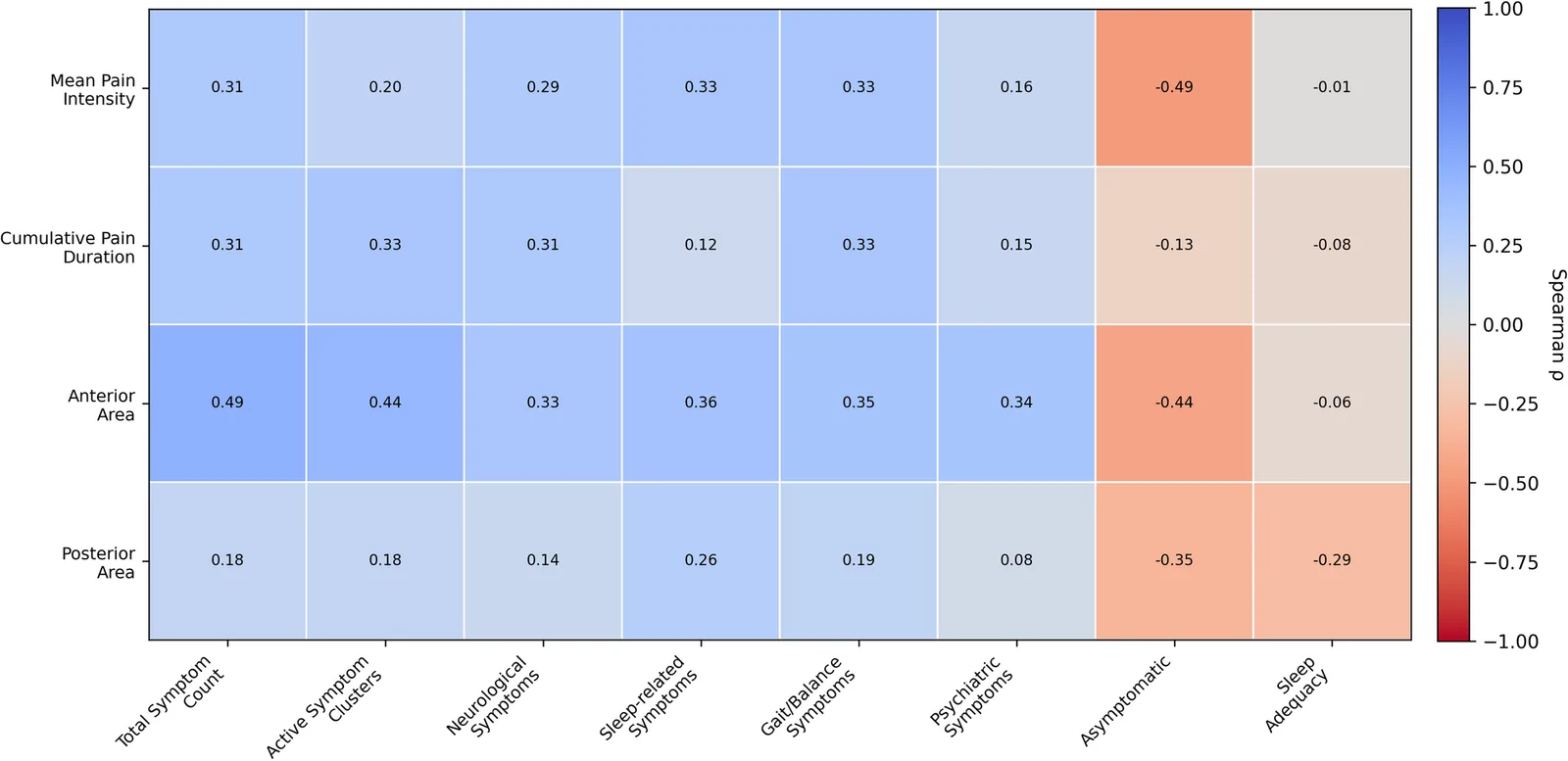
Correlation matrix between pain dimensions and symptom domains. Spearman correlation coefficients (ρ) between pain metrics (mean pain intensity, cumulative pain duration, and spatial extent) and symptom-related variables, including total symptom count, number of active symptom clusters, and domain-specific symptoms (neurological, sleep-related, equilibrium/gait, psychiatric, and asymptomatic). Distinct domain-specific patterns were observed. Neurological and equilibrium/gait symptoms showed consistent associations with both intensity and cumulative/spatial measures, suggesting a global contribution to pain expression. Psychiatric symptoms were primarily associated with spatial extent, indicating a selective influence on pain distribution. Sleep-related symptoms were associated with pain intensity and spatial extent, but not with cumulative duration, indicating a dissociation between perceived severity and persistence. The asymptomatic domain demonstrated consistent negative correlations across all pain metrics, supporting internal dataset validity

Weak to moderate positive correlations emerged between total symptom count and mean pain intensity (ρ = 0.31, *p* = 0.009), cumulative duration (ρ = 0.31, *p* = 0.011), and anterior spatial extent (ρ = 0.49, *p* < 0.001). The number of active symptom clusters was correlated with cumulative duration (ρ = 0.33, *p* = 0.007) and anterior spatial extent (ρ = 0.44, *p* < 0.001), but not with mean pain intensity. Neurological symptoms showed weak to moderate correlations with intensity (ρ = 0.29, *p* = 0.018), duration (ρ = 0.31, *p* = 0.010), and anterior spatial extent (ρ = 0.33, *p* = 0.006). Equilibrium and gait-related symptoms showed associations with cumulative duration (ρ = 0.33, *p* = 0.005), anterior spatial extent (ρ = 0.35, *p* = 0.003), and mean pain intensity (ρ = 0.33, *p* = 0.006). Psychiatric symptoms were primarily associated with anterior spatial extent (ρ = 0.34, *p* = 0.004), with weaker or non-significant associations with other pain metrics. Sleep-related symptoms were correlated with mean pain intensity (ρ = 0.33, *p* = 0.006) and spatial extent (anterior: ρ = 0.36, *p* = 0.002; posterior: ρ = 0.26, *p* = 0.031), but not with cumulative duration. Sleep adequacy was not significantly associated with most pain metrics, except for a weak negative correlation with posterior spatial extent (ρ = −0.29, *p* = 0.018). The asymptomatic domain showed consistent negative correlations with all pain metrics, including mean pain intensity (ρ = −0.49, *p* < 0.001), anterior spatial extent (ρ = −0.44, *p* < 0.001), and posterior spatial extent (ρ = −0.35, *p* = 0.003), indicating lower pain burden in the absence of reported symptoms (Fig. 2). Additional symptom domains, including respiratory, cardiovascular, and dermatological symptoms, were also explored. Despite their relatively high prevalence in the cohort, these domains showed weak and inconsistent correlations with pain metrics. Respiratory symptoms demonstrated negligible associations with mean pain intensity (ρ = −0.01), cumulative duration (ρ = −0.03), and spatial extent (anterior: ρ = 0.17; posterior: ρ = 0.01). Similarly, cardiovascular symptoms exhibited weak correlations across all pain dimensions (ρ ranging from − 0.01 to 0.11). Dermatological symptoms showed slightly higher but still weak associations with pain metrics (ρ ranging from 0.11 to 0.19), without a consistent pattern across dimensions. These findings indicate that, although prevalent, these domains were not meaningfully associated with the multidimensional structure of pain in this cohort.

### Correlation of pain metrics with medication variables

Total medication frequency showed no significant correlation with mean pain intensity (ρ = 0.07, *p* = 0.552), but was weakly correlated with cumulative duration (ρ = 0.30, *p* = 0.013) and posterior spatial extent (ρ = 0.32, *p* = 0.008). The number of medication classes used showed a moderate correlation with cumulative duration (ρ = 0.40, *p* < 0.001) and a weak correlation with anterior spatial extent (ρ = 0.27, *p* = 0.028), but was not associated with mean pain intensity. Therapeutic complexity demonstrated a moderate correlation with cumulative duration (ρ = 0.46, *p* < 0.001) and a weaker correlation with anterior spatial extent (ρ = 0.32, *p* = 0.008), without significant association with mean pain intensity.

### Correlation of pain metrics with crisis variables

**Fig. 3:**
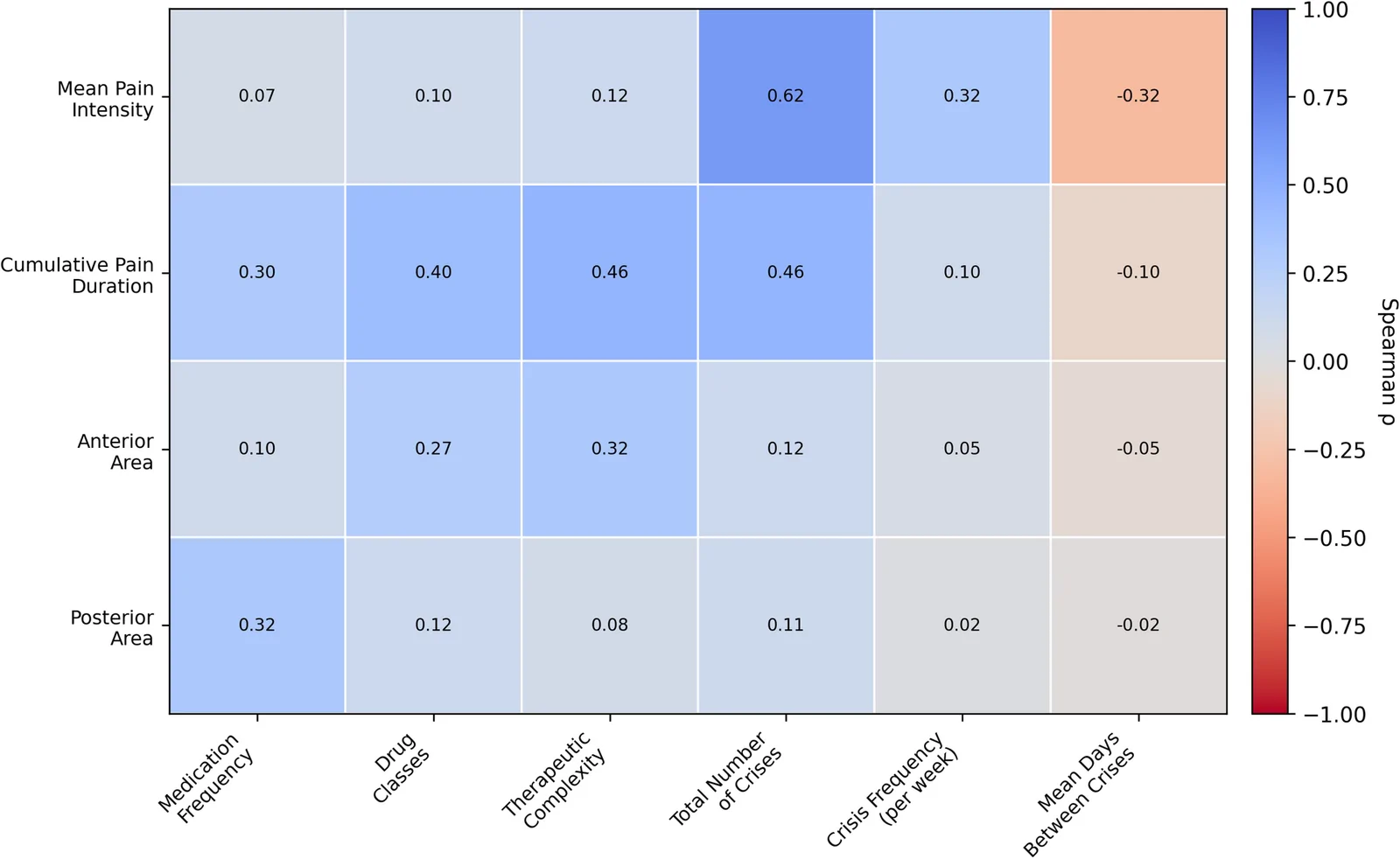
Correlation matrix of pain dimensions, medication-related variables, and crisis-related measures. Spearman correlation coefficients (ρ) between pain metrics, medication-related variables (frequency, number of drug classes, and therapeutic complexity), and crisis-related measures (frequency of crises, total number of crises, and mean days between crises). Crisis variables were primarily associated with mean pain intensity, with limited or no association with spatial extent and only partial association with cumulative duration, supporting their role as markers of episodic pain dynamics. In contrast, medication-related variables were consistently associated with cumulative pain duration and, to a lesser extent, spatial extent, but not with mean pain intensity, indicating that treatment complexity is more closely aligned with persistent and widespread pain burden rather than episodic intensity

Frequency of pain crises per week showed a weak positive correlation with mean pain intensity (ρ = 0.32, *p* = 0.047), but was not significantly associated with cumulative duration or spatial extent. Mean days between crises showed a weak negative correlation with mean pain intensity (ρ = −0.32, *p* = 0.047), with no significant associations with cumulative duration or spatial extent. The total number of crises demonstrated a moderate correlation with mean pain intensity (ρ = 0.62, *p* < 0.001) and cumulative duration (ρ = 0.46, *p* = 0.003), but was not significantly associated with spatial extent (Fig. 3).

## Discussion

Our results indicate that cumulative pain duration and spatial distribution were the factors most associated with between-group differences, with patients in the high-burden profile exhibiting greater cumulative pain duration and spatial extent compared to the low-burden profile, while differences in mean pain intensity were smaller. This finding aligns with previous work showing that pain in PD is multidimensional (Ford 2010; Wasner and Deuschl 2012). Cumulative duration reflects temporal persistence and may better capture overall symptom burden and its impact on daily functioning (Von Korff et al. 1992). Patients in the high-burden profile not only reported greater pain intensity but also experienced pain over longer cumulative durations and across wider body distribution, suggesting a distinct subgroup with implications for tailored clinical management, consistent with prior descriptions of heterogeneity in Parkinson’s disease (Mylius et al. 2021).

An important methodological consideration in mobile health studies is potential engagement bias. Our analysis showed that the point-biserial correlation between days of activity and group membership was weak and non-significant (*r* = 0.16, *p* = 0.20), with both groups demonstrating sustained reporting activity (high-burden: 157 ± 92 days; low-burden: 112 ± 85 days). Thus, differences in reporting frequency were unlikely to account for observed between-group differences. Descriptive classification based on amplitude identified duration and spatial distribution as main contributors to group separation (Fisher 1936; Rousseeuw 1987; Jain 2010). Although variables operate on different scales, the pattern was consistent showing cumulative pain duration differed by 6.7-fold and spatial extent by 9- to 11-fold, compared to a 2.4-fold difference in mean pain intensity. Average pain intensity represents an average summary of pain severity over time (Stone et al. 2004), compressing temporal and spatial variability into a single central value. In contrast, cumulative parameters capture how pain persists and is distributed, representing total pain burden rather than average severity (Melzack and Casey 1968; Turk and Melzack 2011).

Correlation analyses refined this interpretation. Mean pain intensity was moderately associated with cumulative duration (ρ = 0.66) and spatial extent (ρ = 0.60 anterior; ρ = 0.44 posterior), indicating partial reflection of a shared underlying dimension. The weak correlation between mean intensity and mean duration (ρ = 0.15, *p* = 0.23) demonstrates that intensity does not capture the temporal structure of pain episodes. The dissociation is supported by the finding that mean pain duration per episode was not associated with spatial extent, while remaining moderately associated with cumulative duration, indicating distinct aspects of pain organization. Patients with similar average pain intensity showed greater differences in persistence and spatial distribution. Thus, average intensity captures only part of the total pain burden and should complement cumulative and spatial measures. Because average pain intensity reflects central tendency and varies within a relatively narrow range, it may be less sensitive to capturing variations in overall pain burden over time, particularly when temporal and spatial variability are aggregated into a single central measure (Stone et al. 2012; Schneider et al. 2013), and therefore less informative for assessing treatment effects (Turk and Melzack 2011; Dworkin et al. 2005). Reduced sensitivity should not be interpreted as a limitation of pain intensity as a construct, but rather as a consequence of how it is synthesized. This distinction is particularly relevant in chronic fluctuating conditions like Parkinson’s disease, where symptoms evolve over time and involve multiple overlapping mechanisms (Chaudhuri et al. 2006; Wasner and Deuschl 2012). Patients with a high-burden profile experienced pain that persisted longer and affected larger body areas, potentially reflecting central pain processing mechanisms or multiple physiological systems (Apkarian et al. 2009; Tracey and Mantyh 2007). Pain burden in Parkinson’s disease is better understood as an expansion of temporal and spatial dimensions rather than a proportional increase in intensity alone.

This observation may also help explain why pain intervention trials in Parkinson’s disease have frequently reported modest or inconsistent benefits despite considerable efforts to improve pain management (Karnik et al. 2020). Although several methodological and clinical factors likely contribute to this challenge, our findings suggest that clinically meaningful improvements in overall pain burden may not always be fully reflected by changes in mean pain intensity alone. Pain duration and spatial distribution have previously been recognized as important components of pain assessment in Parkinson’s disease (Perez-Lloret et al. 2016). However, our findings further suggest that these dimensions are not merely complementary descriptors but major determinants of overall pain burden. Consequently, therapeutic benefits associated with reductions in cumulative pain duration or spatial extent may be only partially reflected when treatment response is summarized primarily by average pain intensity.

The crisis-related analyses showed a distinct pattern: variables related to pain episodes were associated with mean pain intensity, with limited or no association with cumulative duration and spatial extent. Pain crises represent an episodic temporal dimension of pain, closely linked to intensity but largely independent of spatial distribution. The total number of crises demonstrated a moderate association with cumulative duration (ρ = 0.46, *p* = 0.003), indicating that while crises contribute to overall pain accumulation, they do not fully capture continuous pain burden. These findings reinforce the distinction between episodic and continuous components of pain, consistent with prior descriptions of temporal variability in chronic pain conditions (Treede et al. 2019).

Clinically, relying solely on pain intensity may provide an incomplete representation of overall patient burden. Although mean pain intensity discriminated patient groups, the magnitude of between-group differences was substantially greater for cumulative duration and spatial extent. Patients with prolonged or widespread pain may be under-recognized when assessment relies primarily on average intensity-based measures (Chaudhuri and Schapira 2009; Ford 2010; Galve Villa et al. 2021). The high-burden subgroup was characterized not only by higher intensity, but by longer pain duration, wider spatial distribution, and greater medication complexity, in line with previous reports of pain heterogeneity in PD (Broen et al. 2012; Martinez-Martin et al. 2011; Silverdale et al. 2018). The number of medication class count and therapeutic complexity were linked to cumulative pain duration and, to a lesser extent, spatial extent, but not with mean pain intensity. This suggests that treatment burden is more aligned with pain persistence and distribution rather than average severity, consistent with the interpretation that treatment complexity is associated with sustained and widespread pain patterns rather than episodic intensity alone (Turk and Melzack 2011). The overall use of antiparkinson drugs and conventional analgesics was comparable between groups, patients in the high pain burden profile more frequently received tricyclic antidepressants. This finding is consistent with the use of adjuvant pharmacological strategies commonly employed in chronic pain management. Nevertheless, the overall medication profile indicates that the greater therapeutic complexity observed in the high pain burden group was not attributable to a single pharmacological class, but rather to a broader and more complex multimodal treatment approach involving multiple therapeutic strategies. The high-burden profile may benefit from closer monitoring and individualized management (Alis et al. 2025), while the low-burden profile exhibited more localized and intermittent patterns, suggesting different mechanisms across subgroups (Mylius et al. 2021). Sleep adequacy (≥ 6 h) did not differ between groups, but the low overall proportion of adequate sleep highlights sleep as a therapeutic target. Patients in the high-burden profile demonstrated higher multidomain symptom burden, primarily involving neurological and sleep-related symptoms. Prior studies indicate that pain in Parkinson’s disease rarely occurs in isolation but as part of a broader non-motor symptom spectrum (Chaudhuri et al. 2011), including fatigue, sleep disturbances, and mood-related symptoms (Antonini et al. 2012; Martinez-Martin et al. 2011). Notably, associations between pain metrics and symptom domains were stronger for cumulative and spatial measures than for mean intensity.

Mean pain intensity was associated with total number of reported symptoms but not with number of active symptom domains, suggesting that increases in pain intensity are primarily related to within-domain symptom amplification rather than broader multidomain involvement (Melzack 1999; Turk and Melzack 2011; Fillingim et al. 2009). Neurological symptoms showed associations with intensity, duration, and spatial extent, suggesting global contribution to pain expression, consistent with evidence linking pain to central nervous system dysfunction (Ford 2010; Wasner and Deuschl 2012). Balance and gait symptoms were associated with cumulative duration and spatial extent, aligning with musculoskeletal and postural components where axial rigidity and impaired control contribute to sustained generalized pain (Beiske et al. 2009; Defazio et al. 2008). Psychiatric symptoms were associated with spatial extent (ρ = 0.34, *p* = 0.004), suggesting selective influence on pain distribution rather than severity (Villemure and Bushnell 2009; Wiech and Tracey 2009). Sleep-related symptoms were associated with pain intensity and spatial extent but not cumulative duration, while sleep adequacy showed minimal associations, suggesting that quantitative sleep duration may not capture aspects of sleep relevant to pain experience (Finan et al. 2013; Smith et al. 2007). Respiratory, cardiovascular, and dermatological symptoms showed weak inconsistent associations, indicating that symptom prevalence alone does not imply relevance to pain burden. Finally, the asymptomatic domain showed consistent negative correlations across all pain metrics, corroborating to internal validity.

The use of longitudinal mobile health data introduces both strengths and limitations. Variation in follow-up duration reflects real-world patterns of app use and engagement. Cumulative measures may reflect both symptom burden and recording behavior, but our engagement analysis showed no association between recording frequency and cluster membership, mitigating this concern. These measures should be interpreted as indicators of recorded burden rather than absolute exposure. This variability provides insight into real-world symptom monitoring, corroborating previous mobile health studies in Parkinson’s disease (Linares-Del Rey et al. 2019; Bougea 2025).

This study has several limitations. Selection and engagement bias may limit generalizability. The modest sample size may affect clustering stability, although used descriptively. Limitations include derived variables, absence of standardized clinical staging, lack of systematically collected comorbidity data, and reliance on patient-reported outcomes without objective validation. The observational design prevents causal inference. Despite these limitations, consistency across dimensions supports robustness of identified profiles. Future studies should focus on external validation, prospective evaluation of profile-guided interventions, integration with objective measures such as wearable sensors, and longitudinal analysis of transitions between profiles (Mammen et al. 2025; Xu et al. 2025).

## Data Availability

Anonymized data may be made available from the corresponding author upon reasonable request and subject to institutional ethics approval.
